# Supplementary material for: Minimal system for assembly of SARS-CoV-2 virus like particles
Source: Sci Rep. 2020 Dec 14;10:21877. doi: 10.1038/s41598-020-78656-w (PMC7736577; doi:10.1038/s41598-020-78656-w)
Supplement: Supplementary file 1 — Supplementary Information 1. [file 41598_2020_78656_MOESM1_ESM.docx]

SUPPLEMENTARY INFORMATION

FOR

**MINIMAL SYSTEM FOR ASSEMBLY OF SARS-COV-2 VIRUS LIKE PARTICLES**

**Authors:**

Heather Swann1,2*, Abhimanyu Sharma2*, Benjamin Preece1,2, Abby Peterson1,2, Crystal Eldredge1,2, David M. Belnap,3,4

Michael Vershinin1,2,3,ϯ and Saveez Saffarian1,2,3,ϯ

**Affiliations:**

1 Center for Cell and Genome Science, University of Utah

2 Department of Physics and Astronomy, University of Utah

3 School of Biological Sciences, University of Utah

4 Department of Biochemistry, University of Utah

ϯ Corresponding authors: [Vershinin@Physics.utah.edu](mailto:Vershinin@Physics.utah.edu) and [Saffarian@Physics.Utah.edu](mailto:Saffarian@Physics.Utah.edu)

*These authors contributed equally to this work

Materials and Methods

Cell Culture:

Cells from the human embryonic kidney 293T cell line were maintained in T-25 flasks using TrypLE Express Enzyme (Gibco) and DMEM with L-Glutamine, 4.5g/L glucose and sodium pyruvate (Corning) supplemented with 10% fetal bovine serum (Gibco). The cells were incubated at 37°C. Once over 90% confluent, the 293T cells were plated onto 100 mm tissue culture dishes with 10 mL of medium which would be ready for transfection the next day.

Transfection and VLP harvest:

When the 293T cells were at 50% confluent in the 100 mm dishes, they were co-transfected with 29.5 µg of humanized membrane in pCDNA3.1, 5.9 µg of humanized spike in pCDNA3.1, and 5.9 µg of humanized envelope in pCDNA3.1 (GenScript). This transfection was carried out using 600 mL of Opti-MEM Reduced Serum Medium (Gibco) and 40 mL of Lipofectamine 2000 (Thermo Fisher Scientific). The cells were incubated at 37°C in 9 mL of Full DMEM with FBS supplementation for 24 hours.

Western Blot protocol:

The specificity of the western blot antibodies are tested as shown in Figure S. the detailed protocol for western blot analysis is below:

1. Add sample buffer to the harvest. Add it in 1:1 ratio, i.e., 10ul sample+10ul buffer

2. Heat at 95°C for 5mins, while waiting set the gel.

3. After 5mins, quick spin the sample and start loading the gel

4. Run gel with running buffer at 90volt for 5mins and then at 160volt for 40mins or till the sample reaches the bottom of the gel

5. Prepare membrane and activate the PVDF membrane by methanol for 5mins. Put the foam and gel-blotting paper in transfect buffer for 5min. (2 foams and 4 blotting papers per gel)

6. Take out the gel and put it in transfer buffer

7. Wash the membrane with transfer buffer once before assembly

8. After you make the assembly stack run with transfer buffer at 35 volt (per gel) for 60mins

9. After the run, place the gel in a box with 7ml blocking buffer for 1hr

10. Add primary antibody with required dilution in 7 ml blocking buffer

11. Incubate overnight

12. Wash with PBS-Tween (2 times 15mins each)

13. Then add secondary antibody 1:20000. So, 0.25ul into 5ml of blocking buffer and keep for

45mins-1hr max and then again wash it with PBS-Tween (2 times 15mins each) and then with

15mins with PBS

14. Then visualize

Imaging:

Licor Odyssey – I would need to double check the model if we need that information but I believe it is model 9120. You are correct that we image at 700 and 800 nm channels. We imaged at 0.0 mm focus offset. The image in Figure 1 was imaged at “highest quality” setting


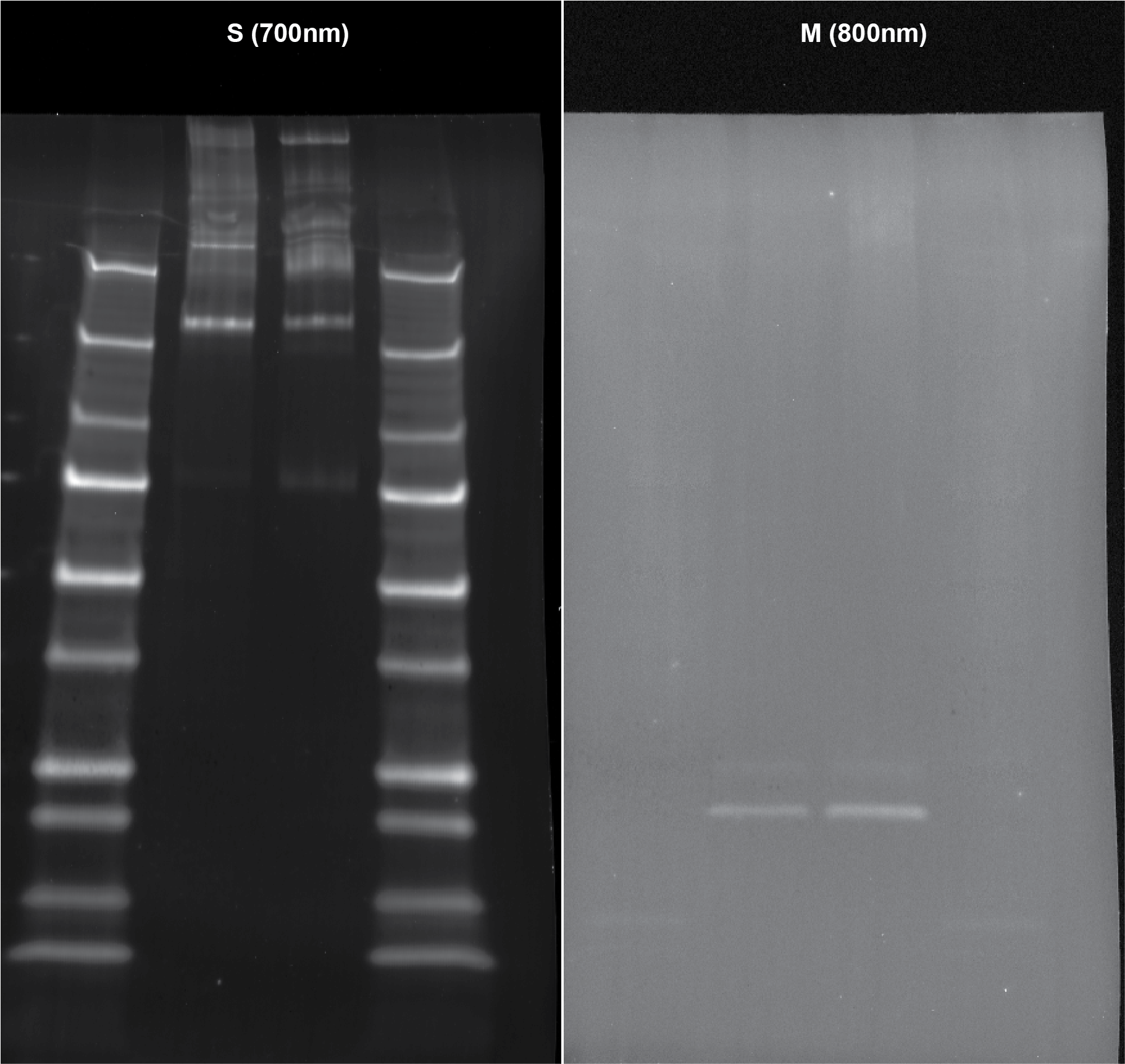


**Figure S1. The data in Fig. 1B with channels separated.** Here, no cropping or contrast adjustment was done. The original 16-bit TIF format images were resaved in 8-bit PNG format using ImageJ (NIH) without compression. Imaging channel labels were added for clarity.


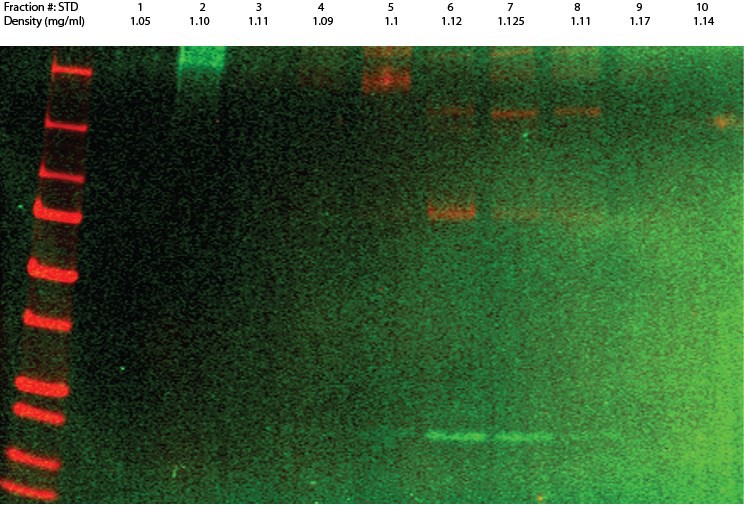


**Figure S2. Sucrose fractionation of VLPs.** The gel was performed simultaneously, scanned at 700 nm and 800 nm, then superimposed after minimal contrast enhancement. Protein size standards (leftmost lane) are same as Fig. 1B.

**
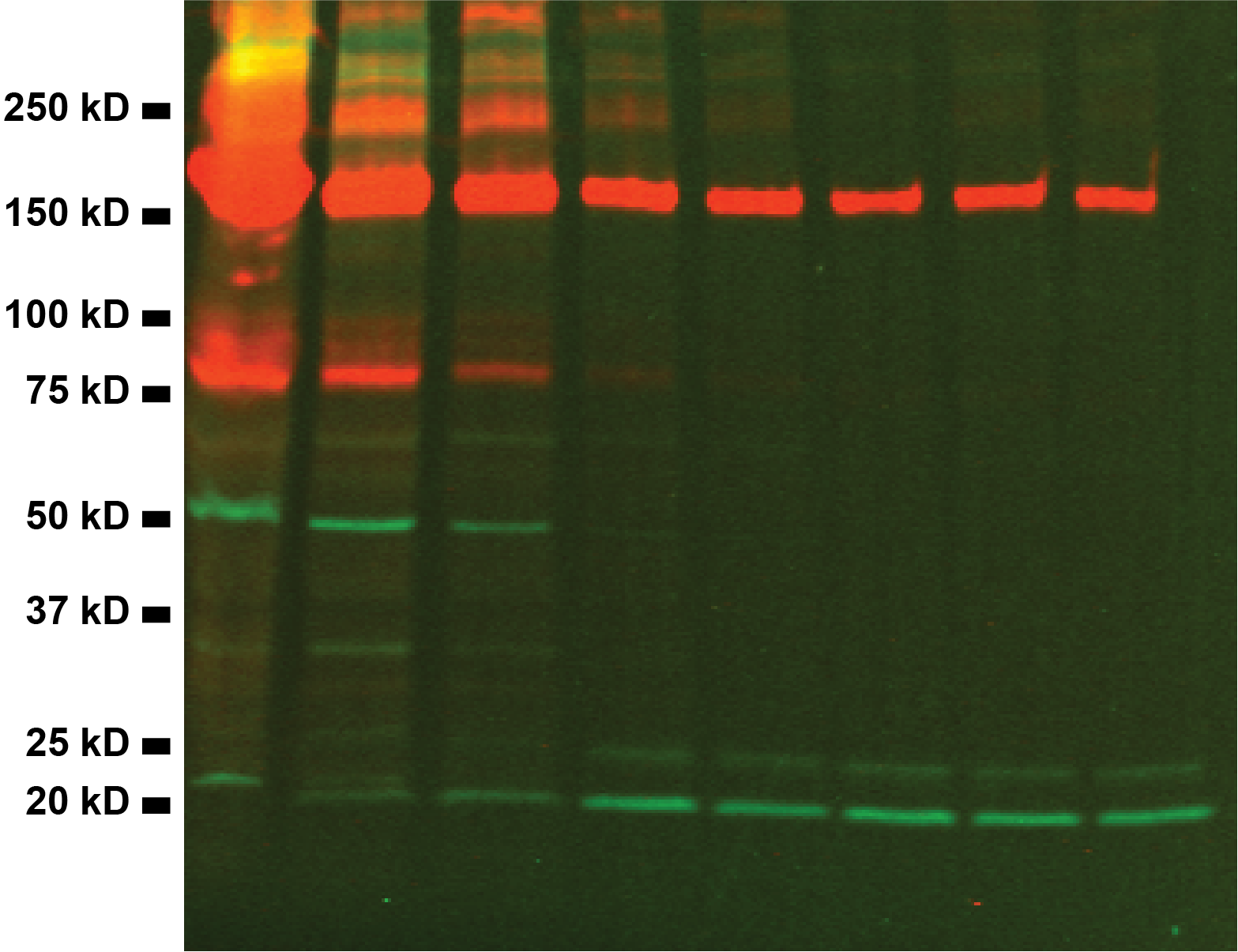
**

**Figure S3. Serial 1:2 dilution analysis of cells.** Both M (green) and S (red) proteins show molecular weight bands above their respective monomer bands (likely representative of aggregation) at highest concentrations. Monomer bands rapidly become dominant at lower dilutions.

**
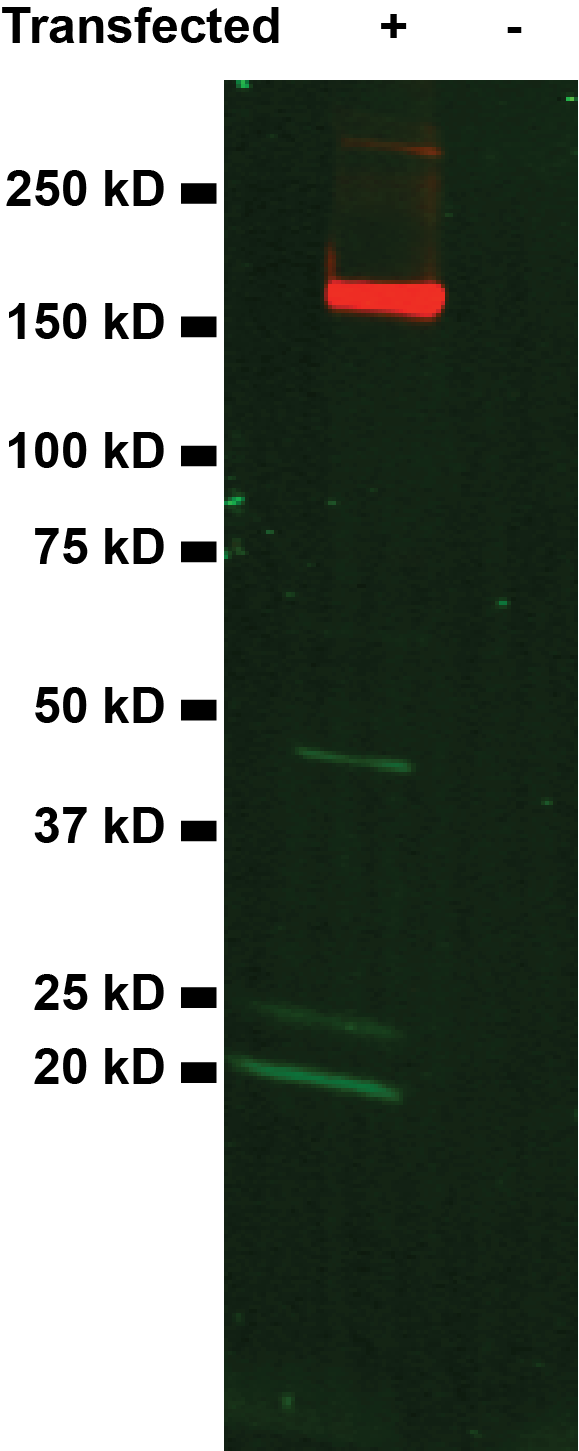
**

**Figure S4. Anti-S and Anti-M antibodies are highly specific.** Transfected cells show M and S presence via antibody probing however un-transfected cells show no bands under identical probing. Unrelated lanes are blanked out for clarity. The gel was performed simultaneously, scanned at 700 nm and 800 nm, then superimposed after minimal contrast enhancement. Protein size standards (leftmost lane) are same as Fig. 1B.


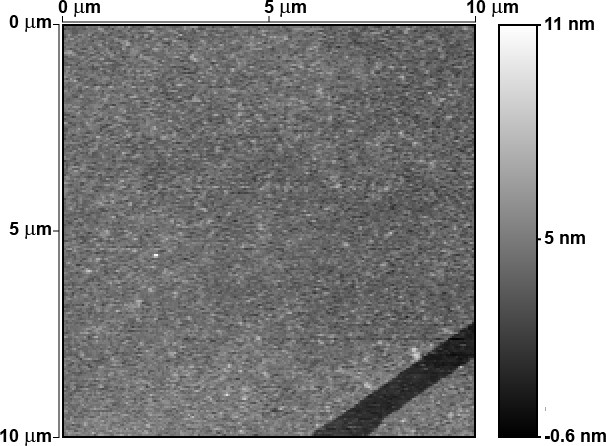


**Figure S5. AFM imaging of glass surface functionalized with anti-S antibody.** Glass surface functionalized as described in main text typically has large flat debris-free areas with occasional shallow step edges. These latter features are only seen after antibody adsorption and therefore correspond to sub-monolayer antibody coverage.


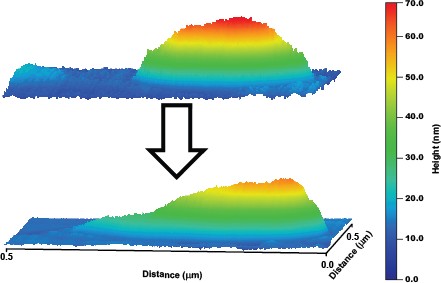


**Figure S6. AFM imaging and bursting of a VLP.** Several repeated scans at 1 nN peak force lead intact VLP (top) to burst (bottom).

Humanized S protein expression plasmid:

GACGGATCGGGAGATCTCCCGATCCCCTATGGTGCACTCTCAGTACAATCTGCTCTGATGCCGCATAGTTAAGCCAGTATCTGCTCCC TGCTTGTGTGTTGGAGGTCGCTGAGTAGTGCGCGAGCAAAATTTAAGCTACAACAAGGCAAGGCTTGACCGACAATTGCATGAAGA ATCTGCTTAGGGTTAGGCGTTTTGCGCTGCTTCGCGAGTACATTTATATTGGCTCATGTCCAATATGACCGCCATGTTGACATTGATTA TTGACTAGTTATTAATAGTAATCAATTACGGGGTCATTAGTTCATAGCCCATATATGGAGTTCCGCGTTACATAACTTACGGTAAATGG CCCGCCTGGCTGACCGCCCAACGACCCCCGCCCATTGACGTCAATAATGACGTATGTTCCCATAGTAACGCCAATAGGGACTTTCCAT TGACGTCAATGGGTGGAGTATTTACGGTAAACTGCCCACTTGGCAGTACATCAAGTGTATCATATGCCAAGTCCGCCCCCTATTGACG TCAATGACGGTAAATGGCCCGCCTGGCATTATGCCCAGTACATGACCTTACGGGACTTTCCTACTTGGCAGTACATCTACGTATTAGT CATCGCTATTACCATGGTGATGCGGTTTTGGCAGTACACCAATGGGCGTGGATAGCGGTTTGACTCACGGGGATTTCCAAGTCTCCAC CCCATTGACGTCAATGGGAGTTTGTTTTGGCACCAAAATCAACGGGACTTTCCAAAATGTCGTAATAACCCCGCCCCGTTGACGCAAA TGGGCGGTAGGCGTGTACGGTGGGAGGTCTATATAAGCAGAGCTCGTTTAGTGAACCGTCAGATCCTCACTCTCTTCCGCATCGCTG TCTGCGAGGGCCAGCTGTTGGGCTCGCGGTTGAGGACAAACTCTTCGCGGTCTTTCCAGTACTCTTGGATCGGAAACCCGTCGGCCT CCGAACGGTACTCCGCCACCGAGGGACCTGAGCGAGTCCGCATCGACCGGATCGGAAAACCTCTCGAGAAAGGCGTCTAACCAGTC ACAGTCGCAAGGTAGGCTGAGCACCGTGGCGGGCGGCAGCGGGTGGCGGTCGGGGTTGTTTCTGGCGGAGGTGCTGCTGATGATG TAATTAAAGTAGGCGGTCTTGAGACGGCGGATGGTCGAGGTGAGGTGTGGCAGGCTTGAGATCCAGCTGTTGGGGTGAGTACTCCC TCTCAAAAGCGGGCATTACTTCTGCGCTAAGATTGTCAGTTTCCAAAAACGAGGAGGATTTGATATTCACCTGGCCCGATCTGGCCAT ACACTTGAGTGACAATGACATCCACTTTGCCTTTCTCTCCACAGGTGTCCACTCCCAGGTCCAAGTTTAAACTTTAATACGACTCACTAT AGGGGCCGCCACCAAGCTTNNNNNGGTACCNNNNNATGTTTGTGTTCCTGGTGCTGCTGCCACTGGTGTCCAGCCAGTGTGTGAAC CTGACCACCAGGACCCAACTTCCTCCTGCCTACACCAACTCCTTCACCAGGGGAGTCTACTACCCTGACAAGGTGTTCAGGTCCTCTGT GCTGCACAGCACCCAGGACCTGTTCCTGCCATTCTTCAGCAATGTGACCTGGTTCCATGCCATCCATGTGTCTGGCACCAATGGCACC AAGAGGTTTGACAACCCTGTGCTGCCATTCAATGATGGAGTCTACTTTGCCAGCACAGAGAAGAGCAACATCATCAGGGGCTGGATT TTTGGCACCACCCTGGACAGCAAGACCCAGTCCCTGCTGATTGTGAACAATGCCACCAATGTGGTGATTAAGGTGTGTGAGTTCCAG TTCTGTAATGACCCATTCCTGGGAGTCTACTACCACAAGAACAACAAGTCCTGGATGGAGTCTGAGTTCAGGGTCTACTCCTCTGCCA ACAACTGTACCTTTGAATATGTGAGCCAACCATTCCTGATGGACTTGGAGGGCAAGCAGGGCAACTTCAAGAACCTGAGGGAGTTTG TGTTCAAGAACATTGATGGCTACTTCAAGATTTACAGCAAACACACACCAATCAACCTGGTGAGGGACCTGCCACAGGGCTTCTCTGC CTTGGAACCACTGGTGGACCTGCCAATTGGCATCAACATCACCAGGTTCCAGACCCTGCTGGCTCTGCACAGGTCCTACCTGACACCT GGAGACTCCTCCTCTGGCTGGACAGCAGGAGCAGCAGCCTACTATGTGGGCTACCTCCAACCAAGGACCTTCCTGCTGAAATACAAT GAGAATGGCACCATCACAGATGCTGTGGACTGTGCCCTGGACCCACTGTCTGAGACCAAGTGTACCCTGAAATCCTTCACAGTGGAG AAGGGCATCTACCAGACCAGCAACTTCAGGGTCCAACCAACAGAGAGCATTGTGAGGTTTCCAAACATCACCAACCTGTGTCCATTTG GAGAGGTGTTCAATGCCACCAGGTTTGCCTCTGTCTATGCCTGGAACAGGAAGAGGATTAGCAACTGTGTGGCTGACTACTCTGTGC TCTACAACTCTGCCTCCTTCAGCACCTTCAAGTGTTATGGAGTGAGCCCAACCAAACTGAATGACCTGTGTTTCACCAATGTCTATGCT GACTCCTTTGTGATTAGGGGAGATGAGGTGAGACAGATTGCCCCTGGACAAACAGGCAAGATTGCTGACTACAACTACAAACTGCCT GATGACTTCACAGGCTGTGTGATTGCCTGGAACAGCAACAACCTGGACAGCAAGGTGGGAGGCAACTACAACTACCTCTACAGACTG TTCAGGAAGAGCAACCTGAAACCATTTGAGAGGGACATCAGCACAGAGATTTACCAGGCTGGCAGCACACCATGTAATGGAGTGGA

GGGCTTCAACTGTTACTTTCCACTCCAATCCTATGGCTTCCAACCAACCAATGGAGTGGGCTACCAACCATACAGGGTGGTGGTGCTG TCCTTTGAACTGCTCCATGCCCCTGCCACAGTGTGTGGACCAAAGAAGAGCACCAACCTGGTGAAGAACAAGTGTGTGAACTTCAAC TTCAATGGACTGACAGGCACAGGAGTGCTGACAGAGAGCAACAAGAAGTTCCTGCCATTCCAACAGTTTGGCAGGGACATTGCTGA CACCACAGATGCTGTGAGGGACCCACAGACCTTGGAGATTCTGGACATCACACCATGTTCCTTTGGAGGAGTGTCTGTGATTACACCT GGCACCAACACCAGCAACCAGGTGGCTGTGCTCTACCAGGATGTGAACTGTACTGAGGTGCCTGTGGCTATCCATGCTGACCAACTT ACACCAACCTGGAGGGTCTACAGCACAGGCAGCAATGTGTTCCAGACCAGGGCTGGCTGTCTGATTGGAGCAGAGCATGTGAACAA CTCCTATGAGTGTGACATCCCAATTGGAGCAGGCATCTGTGCCTCCTACCAGACCCAGACCAACAGCCCAAGGAGGGCAAGGTCTGT GGCAAGCCAGAGCATCATTGCCTACACAATGAGTCTGGGAGCAGAGAACTCTGTGGCTTACAGCAACAACAGCATTGCCATCCCAAC CAACTTCACCATCTCTGTGACCACAGAGATTCTGCCTGTGAGTATGACCAAGACCTCTGTGGACTGTACAATGTATATCTGTGGAGAC AGCACAGAGTGTAGCAACCTGCTGCTCCAATATGGCTCCTTCTGTACCCAACTTAACAGGGCTCTGACAGGCATTGCTGTGGAACAG GACAAGAACACCCAGGAGGTGTTTGCCCAGGTGAAGCAGATTTACAAGACACCTCCAATCAAGGACTTTGGAGGCTTCAACTTCAGC CAGATTCTGCCTGACCCAAGCAAGCCAAGCAAGAGGTCCTTCATTGAGGACCTGCTGTTCAACAAGGTGACCCTGGCTGATGCTGGC TTCATCAAGCAATATGGAGACTGTCTGGGAGACATTGCTGCCAGGGACCTGATTTGTGCCCAGAAGTTCAATGGACTGACAGTGCTG CCTCCACTGCTGACAGATGAGATGATTGCCCAATACACCTCTGCCCTGCTGGCTGGCACCATCACCTCTGGCTGGACCTTTGGAGCAG GAGCAGCCCTCCAAATCCCATTTGCTATGCAGATGGCTTACAGGTTCAATGGCATTGGAGTGACCCAGAATGTGCTCTATGAGAACC AGAAACTGATTGCCAACCAGTTCAACTCTGCCATTGGCAAGATTCAGGACTCCCTGTCCAGCACAGCCTCTGCCCTGGGCAAACTCCA AGATGTGGTGAACCAGAATGCCCAGGCTCTGAACACCCTGGTGAAGCAACTTTCCAGCAACTTTGGAGCCATCTCCTCTGTGCTGAAT GACATCCTGAGCAGACTGGACAAGGTGGAGGCTGAGGTCCAGATTGACAGACTGATTACAGGCAGACTCCAATCCCTCCAAACCTAT GTGACCCAACAACTTATCAGGGCTGCTGAGATTAGGGCATCTGCCAACCTGGCTGCCACCAAGATGAGTGAGTGTGTGCTGGGACA AAGCAAGAGGGTGGACTTCTGTGGCAAGGGCTACCACCTGATGAGTTTTCCACAGTCTGCCCCTCATGGAGTGGTGTTCCTGCATGT GACCTATGTGCCTGCCCAGGAGAAGAACTTCACCACAGCCCCTGCCATCTGCCATGATGGCAAGGCTCACTTTCCAAGGGAGGGAGT GTTTGTGAGCAATGGCACCCACTGGTTTGTGACCCAGAGGAACTTCTATGAACCACAGATTATCACCACAGACAACACCTTTGTGTCT GGCAACTGTGATGTGGTGATTGGCATTGTGAACAACACAGTCTATGACCCACTCCAACCTGAACTGGACTCCTTCAAGGAGGAACTG GACAAATACTTCAAGAACCACACCAGCCCTGATGTGGACCTGGGAGACATCTCTGGCATCAATGCCTCTGTGGTGAACATCCAGAAG GAGATTGACAGACTGAATGAGGTGGCTAAGAACCTGAATGAGTCCCTGATTGACCTCCAAGAACTGGGCAAATATGAACAATACATC AAGTGGCCATGGTACATCTGGCTGGGCTTCATTGCTGGACTGATTGCCATTGTGATGGTGACCATAATGCTGTGTTGTATGACCTCCT GTTGTTCCTGTCTGAAAGGCTGTTGTTCCTGTGGCTCCTGTTGTAAGTTTGATGAGGATGACTCTGAACCTGTGCTGAAAGGAGTGAA ACTGCACTACACCTGANNNNNTCTAGANNNNNGCGGCCGCCGAATTCGGGCCCGTTTAAACCCGCTGATCAGCCTCGACTGTGCCT TCTAGTTGCCAGCCATCTGTTGTTTGCCCCTCCCCCGTGCCTTCCTTGACCCTGGAAGGTGCCACTCCCACTGTCCTTTCCTAATAAAAT GAGGAAATTGCATCGCATTGTCTGAGTAGGTGTCATTCTATTCTGGGGGGTGGGGTGGGGCAGGACAGCAAGGGGGAGGATTGGG AAGACAATAGCAGGCATGCTGGGGATGCGGTGGGCTCTATGGCTTCTGAGGCGGAAAGAACCAGCTGGGGCTCTAGGGGGTATCC CCACGCGCCCTGTAGCGGCGCATTAAGCGCGGCGGGTGTGGTGGTTACGCGCAGCGTGACCGCTACACTTGCCAGCGCCCTAGCGC CCGCTCCTTTCGCTTTCTTCCCTTCCTTTCTCGCCACGTTCGCCGGCTTTCCCCGTCAAGCTCTAAATCGGGGGCTCCCTTTAGGGTTCC GATTTAGTGCTTTACGGCACCTCGACCCCAAAAAACTTGATTAGGGTGATGGTTCACGTAGTGGGCCATCGCCCTGATAGACGGTTTT

TCGCCCTTTGACGTTGGAGTCCACGTTCTTTAATAGTGGACTCTTGTTCCAAACTGGAACAACACTCAACCCTATCTCGGTCTATTCTTT TGATTTATAAGGGATTTTGCCGATTTCGGCCTATTGGTTAAAAAATGAGCTGATTTAACAAAAATTTAACGCGAATTAATTCTGTGGA ATGTGTGTCAGTTAGGGTGTGGAAAGTCCCCAGGCTCCCCAGCAGGCAGAAGTATGCAAAGCATGCATCTCAATTAGTCAGCAACCA GGTGTGGAAAGTCCCCAGGCTCCCCAGCAGGCAGAAGTATGCAAAGCATGCATCTCAATTAGTCAGCAACCATAGTCCCGCCCCTAA CTCCGCCCATCCCGCCCCTAACTCCGCCCAGTTCCGCCCATTCTCCGCCCCATGGCTGACTAATTTTTTTTATTTATGCAGAGGCCGAGG CCGCCTCTGCCTCTGAGCTATTCCAGAAGTAGTGAGGAGGCTTTTTTGGAGGCCTAGGCTTTTGCAAAAAGCTCCCGGGAGCTTGTAT ATCCATTTTCGGATCTGATCAGCACGTGATGAAAAAGCCTGAACTCACCGCGACGTCTGTCGAGAAGTTTCTGATCGAAAAGTTCGAC AGCGTCTCCGACCTGATGCAGCTCTCGGAGGGCGAAGAATCTCGTGCTTTCAGCTTCGATGTAGGAGGGCGTGGATATGTCCTGCGG GTAAATAGCTGCGCCGATGGTTTCTACAAAGATCGTTATGTTTATCGGCACTTTGCATCGGCCGCGCTCCCGATTCCGGAAGTGCTTG ACATTGGGGAATTCAGCGAGAGCCTGACCTATTGCATCTCCCGCCGTGCACAGGGTGTCACGTTGCAAGACCTGCCTGAAACCGAAC TGCCCGCTGTTCTGCAGCCGGTCGCGGAGGCCATGGATGCGATCGCTGCGGCCGATCTTAGCCAGACGAGCGGGTTCGGCCCATTC GGACCGCAAGGAATCGGTCAATACACTACATGGCGTGATTTCATATGCGCGATTGCTGATCCCCATGTGTATCACTGGCAAACTGTG ATGGACGACACCGTCAGTGCGTCCGTCGCGCAGGCTCTCGATGAGCTGATGCTTTGGGCCGAGGACTGCCCCGAAGTCCGGCACCTC GTGCACGCGGATTTCGGCTCCAACAATGTCCTGACGGACAATGGCCGCATAACAGCGGTCATTGACTGGAGCGAGGCGATGTTCGG GGATTCCCAATACGAGGTCGCCAACATCTTCTTCTGGAGGCCGTGGTTGGCTTGTATGGAGCAGCAGACGCGCTACTTCGAGCGGAG GCATCCGGAGCTTGCAGGATCGCCGCGGCTCCGGGCGTATATGCTCCGCATTGGTCTTGACCAACTCTATCAGAGCTTGGTTGACGG CAATTTCGATGATGCAGCTTGGGCGCAGGGTCGATGCGACGCAATCGTCCGATCCGGAGCCGGGACTGTCGGGCGTACACAAATCG CCCGCAGAAGCGCGGCCGTCTGGACCGATGGCTGTGTAGAAGTACTCGCCGATAGTGGAAACCGACGCCCCAGCACTCGTCCGAGG GCAAAGGAATAGCACGTGCTACGAGATTTCGATTCCACCGCCGCCTTCTATGAAAGGTTGGGCTTCGGAATCGTTTTCCGGGACGCC GGCTGGATGATCCTCCAGCGCGGGGATCTCATGCTGGAGTTCTTCGCCCACCCCAACTTGTTTATTGCAGCTTATAATGGTTACAAAT AAAGCAATAGCATCACAAATTTCACAAATAAAGCATTTTTTTCACTGCATTCTAGTTGTGGTTTGTCCAAACTCATCAATGTATCTTATC ATGTCTGTATACCGTCGACCTCTAGCTAGAGCTTGGCGTAATCATGGTCATAGCTGTTTCCTGTGTGAAATTGTTATCCGCTCACAATT CCACACAACATACGAGCCGGAAGCATAAAGTGTAAAGCCTGGGGTGCCTAATGAGTGAGCTAACTCACATTAATTGCGTTGCGCTCA CTGCCCGCTTTCCAGTCGGGAAACCTGTCGTGCCAGCTGCATTAATGAATCGGCCAACGCGCGGGGAGAGGCGGTTTGCGTATTGG GCGCTCTTCCGCTTCCTCGCTCACTGACTCGCTGCGCTCGGTCGTTCGGCTGCGGCGAGCGGTATCAGCTCACTCAAAGGCGGTAATA CGGTTATCCACAGAATCAGGGGATAACGCAGGAAAGAACATGTGAGCAAAAGGCCAGCAAAAGGCCAGGAACCGTAAAAAGGCCG CGTTGCTGGCGTTTTTCCATAGGCTCCGCCCCCCTGACGAGCATCACAAAAATCGACGCTCAAGTCAGAGGTGGCGAAACCCGACAG GACTATAAAGATACCAGGCGTTTCCCCCTGGAAGCTCCCTCGTGCGCTCTCCTGTTCCGACCCTGCCGCTTACCGGATACCTGTCCGCC TTTCTCCCTTCGGGAAGCGTGGCGCTTTCTCATAGCTCACGCTGTAGGTATCTCAGTTCGGTGTAGGTCGTTCGCTCCAAGCTGGGCT GTGTGCACGAACCCCCCGTTCAGCCCGACCGCTGCGCCTTATCCGGTAACTATCGTCTTGAGTCCAACCCGGTAAGACACGACTTATC GCCACTGGCAGCAGCCACTGGTAACAGGATTAGCAGAGCGAGGTATGTAGGCGGTGCTACAGAGTTCTTGAAGTGGTGGCCTAACT ACGGCTACACTAGAAGAACAGTATTTGGTATCTGCGCTCTGCTGAAGCCAGTTACCTTCGGAAAAAGAGTTGGTAGCTCTTGATCCG GCAAACAAACCACCGCTGGTAGCGGTTTTTTTGTTTGCAAGCAGCAGATTACGCGCAGAAAAAAAGGATCTCAAGAAGATCCTTTGA TCTTTTCTACGGGGTCTGACGCTCAGTGGAACGAAAACTCACGTTAAGGGATTTTGGTCATGAGATTATCAAAAAGGATCTTCACCTA

GATCCTTTTAAATTAAAAATGAAGTTTTAAATCAATCTAAAGTATATATGAGTAAACTTGGTCTGACAGTTACCAATGCTTAATCAGTG AGGCACCTATCTCAGCGATCTGTCTATTTCGTTCATCCATAGTTGCCTGACTCCCCGTCGTGTAGATAACTACGATACGGGAGGGCTTA CCATCTGGCCCCAGTGCTGCAATGATACCGCGAGACCCACGCTCACCGGCTCCAGATTTATCAGCAATAAACCAGCCAGCCGGAAGG GCCGAGCGCAGAAGTGGTCCTGCAACTTTATCCGCCTCCATCCAGTCTATTAATTGTTGCCGGGAAGCTAGAGTAAGTAGTTCGCCAG TTAATAGTTTGCGCAACGTTGTTGCCATTGCTACAGGCATCGTGGTGTCACGCTCGTCGTTTGGTATGGCTTCATTCAGCTCCGGTTCC CAACGATCAAGGCGAGTTACATGATCCCCCATGTTGTGCAAAAAAGCGGTTAGCTCCTTCGGTCCTCCGATCGTTGTCAGAAGTAAGT TGGCCGCAGTGTTATCACTCATGGTTATGGCAGCACTGCATAATTCTCTTACTGTCATGCCATCCGTAAGATGCTTTTCTGTGACTGGT GAGTACTCAACCAAGTCATTCTGAGAATAGTGTATGCGGCGACCGAGTTGCTCTTGCCCGGCGTCAATACGGGATAATACCGCGCCA CATAGCAGAACTTTAAAAGTGCTCATCATTGGAAAACGTTCTTCGGGGCGAAAACTCTCAAGGATCTTACCGCTGTTGAGATCCAGTT CGATGTAACCCACTCGTGCACCCAACTGATCTTCAGCATCTTTTACTTTCACCAGCGTTTCTGGGTGAGCAAAAACAGGAAGGCAAAA TGCCGCAAAAAAGGGAATAAGGGCGACACGGAAATGTTGAATACTCATACTCTTCCTTTTTCAATATTATTGAAGCATTTATCAGGGT TATTGTCTCATGAGCGGATACATATTTGAATGTATTTAGAAAAATAAACAAATAGGGGTTCCGCGCACATTTCCCCGAAAAGTGCCAC CTGACGTC

Humanized M protein expression plasmid:

GACGGATCGGGAGATCTCCCGATCCCCTATGGTGCACTCTCAGTACAATCTGCTCTGATGCCGCATAGTTAAGCCAGTATCTGCTCCC TGCTTGTGTGTTGGAGGTCGCTGAGTAGTGCGCGAGCAAAATTTAAGCTACAACAAGGCAAGGCTTGACCGACAATTGCATGAAGA ATCTGCTTAGGGTTAGGCGTTTTGCGCTGCTTCGCGATGTACGGGCCAGATATACGCGTTGACATTGATTATTGACTAGTTATTAATA GTAATCAATTACGGGGTCATTAGTTCATAGCCCATATATGGAGTTCCGCGTTACATAACTTACGGTAAATGGCCCGCCTGGCTGACCG CCCAACGACCCCCGCCCATTGACGTCAATAATGACGTATGTTCCCATAGTAACGCCAATAGGGACTTTCCATTGACGTCAATGGGTGG AGTATTTACGGTAAACTGCCCACTTGGCAGTACATCAAGTGTATCATATGCCAAGTACGCCCCCTATTGACGTCAATGACGGTAAATG GCCCGCCTGGCATTATGCCCAGTACATGACCTTATGGGACTTTCCTACTTGGCAGTACATCTACGTATTAGTCATCGCTATTACCATGG TGATGCGGTTTTGGCAGTACATCAATGGGCGTGGATAGCGGTTTGACTCACGGGGATTTCCAAGTCTCCACCCCATTGACGTCAATG GGAGTTTGTTTTGGCACCAAAATCAACGGGACTTTCCAAAATGTCGTAACAACTCCGCCCCATTGACGCAAATGGGCGGTAGGCGTG TACGGTGGGAGGTCTATATAAGCAGAGCTCTCTGGCTAACTAGAGAACCCACTGCTTACTGGCTTATCGAAATTAATACGACTCACTA TAGGGAGACCCAAGCTGGCTAGCGCAGCATCTTCAGCTCTTGGGTTTAGCAAAAGTTCATCTCCTTCTGCATCCTTAACTGAGAATGA GCTTTTGTGGGAGCCCACACCAGTCAAGTTGGATTTGAACCCAGCTGCTCTGTACAAGCTTGGTACCGCCACCATGGCCGACAGCAA CGGCACCATCACCGTGGAGGAGCTGAAGAAGCTGCTGGAGCAGTGGAACCTGGTGATCGGCTTCCTGTTCCTGACCTGGATCTGCCT GCTGCAGTTCGCCTACGCCAACAGGAACAGGTTCCTGTACATCATCAAGCTGATCTTCCTGTGGCTGCTGTGGCCCGTGACCCTGGCC TGCTTCGTGCTGGCCGCCGTGTACAGGATCAACTGGATCACCGGCGGCATCGCCATCGCCATGGCCTGCCTGGTGGGCCTGATGTGG CTGAGCTACTTCATCGCCAGCTTCAGGCTGTTCGCCAGGACCAGGAGCATGTGGAGCTTCAACCCCGAGACCAACATCCTGCTGAAC GTGCCCCTGCACGGCACCATCCTGACCAGGCCCCTGCTGGAGAGCGAGCTGGTGATCGGCGCCGTGATCCTGAGGGGCCACCTGAG GATCGCCGGCCACCACCTGGGCAGGTGCGACATCAAGGACCTGCCCAAGGAGATCACCGTGGCCACCAGCAGGACCCTGAGCTACT ACAAGCTGGGCGCCAGCCAGAGGGTGGCCGGCGACAGCGGCTTCGCCGCCTACAGCAGGTACAGGATCGGCAACTACAAGCTGAA

CACCGACCACAGCAGCAGCAGCGACAACATCGCCCTGCTGGTGCAGTAATCTAGAGGGCCCGTTTAAACCCGCTGATCAGCCTCGAC TGTGCCTTCTAGTTGCCAGCCATCTGTTGTTTGCCCCTCCCCCGTGCCTTCCTTGACCCTGGAAGGTGCCACTCCCACTGTCCTTTCCTA ATAAAATGAGGAAATTGCATCGCATTGTCTGAGTAGGTGTCATTCTATTCTGGGGGGTGGGGTGGGGCAGGACAGCAAGGGGGAG GATTGGGAAGACAATAGCAGGCATGCTGGGGATGCGGTGGGCTCTATGGCTTCTGAGGCGGAAAGAACCAGCTGGGGCTCTAGGG GGTATCCCCACGCGCCCTGTAGCGGCGCATTAAGCGCGGCGGGTGTGGTGGTTACGCGCAGCGTGACCGCTACACTTGCCAGCGCC CTAGCGCCCGCTCCTTTCGCTTTCTTCCCTTCCTTTCTCGCCACGTTCGCCGGCTTTCCCCGTCAAGCTCTAAATCGGGGGCTCCCTTTA GGGTTCCGATTTAGTGCTTTACGGCACCTCGACCCCAAAAAACTTGATTAGGGTGATGGTTCACGTAGTGGGCCATCGCCCTGATAG ACGGTTTTTCGCCCTTTGACGTTGGAGTCCACGTTCTTTAATAGTGGACTCTTGTTCCAAACTGGAACAACACTCAACCCTATCTCGGT CTATTCTTTTGATTTATAAGGGATTTTGCCGATTTCGGCCTATTGGTTAAAAAATGAGCTGATTTAACAAAAATTTAACGCGAATTAAT TCTGTGGAATGTGTGTCAGTTAGGGTGTGGAAAGTCCCCAGGCTCCCCAGCAGGCAGAAGTATGCAAAGCATGCATCTCAATTAGTC AGCAACCAGGTGTGGAAAGTCCCCAGGCTCCCCAGCAGGCAGAAGTATGCAAAGCATGCATCTCAATTAGTCAGCAACCATAGTCCC GCCCCTAACTCCGCCCATCCCGCCCCTAACTCCGCCCAGTTCCGCCCATTCTCCGCCCCATGGCTGACTAATTTTTTTTATTTATGCAGA GGCCGAGGCCGCCTCTGCCTCTGAGCTATTCCAGAAGTAGTGAGGAGGCTTTTTTGGAGGCCTAGGCTTTTGCAAAAAGCTCCCGGG AGCTTGTATATCCATTTTCGGATCTGATCAAGAGACAGGATGAGGATCGTTTCGCATGATTGAACAAGATGGATTGCACGCAGGTTCT CCGGCCGCTTGGGTGGAGAGGCTATTCGGCTATGACTGGGCACAACAGACAATCGGCTGCTCTGATGCCGCCGTGTTCCGGCTGTCA GCGCAGGGGCGCCCGGTTCTTTTTGTCAAGACCGACCTGTCCGGTGCCCTGAATGAACTGCAGGACGAGGCAGCGCGGCTATCGTG GCTGGCCACGACGGGCGTTCCTTGCGCAGCTGTGCTCGACGTTGTCACTGAAGCGGGAAGGGACTGGCTGCTATTGGGCGAAGTGC CGGGGCAGGATCTCCTGTCATCTCACCTTGCTCCTGCCGAGAAAGTATCCATCATGGCTGATGCAATGCGGCGGCTGCATACGCTTGA TCCGGCTACCTGCCCATTCGACCACCAAGCGAAACATCGCATCGAGCGAGCACGTACTCGGATGGAAGCCGGTCTTGTCGATCAGGA TGATCTGGACGAAGAGCATCAGGGGCTCGCGCCAGCCGAACTGTTCGCCAGGCTCAAGGCGCGCATGCCCGACGGCGAGGATCTCG TCGTGACCCATGGCGATGCCTGCTTGCCGAATATCATGGTGGAAAATGGCCGCTTTTCTGGATTCATCGACTGTGGCCGGCTGGGTG TGGCGGACCGCTATCAGGACATAGCGTTGGCTACCCGTGATATTGCTGAAGAGCTTGGCGGCGAATGGGCTGACCGCTTCCTCGTGC TTTACGGTATCGCCGCTCCCGATTCGCAGCGCATCGCCTTCTATCGCCTTCTTGACGAGTTCTTCTGAGCGGGACTCTGGGGTTCGAA ATGACCGACCAAGCGACGCCCAACCTGCCATCACGAGATTTCGATTCCACCGCCGCCTTCTATGAAAGGTTGGGCTTCGGAATCGTTT TCCGGGACGCCGGCTGGATGATCCTCCAGCGCGGGGATCTCATGCTGGAGTTCTTCGCCCACCCCAACTTGTTTATTGCAGCTTATAA TGGTTACAAATAAAGCAATAGCATCACAAATTTCACAAATAAAGCATTTTTTTCACTGCATTCTAGTTGTGGTTTGTCCAAACTCATCA ATGTATCTTATCATGTCTGTATACCGTCGACCTCTAGCTAGAGCTTGGCGTAATCATGGTCATAGCTGTTTCCTGTGTGAAATTGTTAT CCGCTCACAATTCCACACAACATACGAGCCGGAAGCATAAAGTGTAAAGCCTGGGGTGCCTAATGAGTGAGCTAACTCACATTAATT GCGTTGCGCTCACTGCCCGCTTTCCAGTCGGGAAACCTGTCGTGCCAGCTGCATTAATGAATCGGCCAACGCGCGGGGAGAGGCGG TTTGCGTATTGGGCGCTCTTCCGCTTCCTCGCTCACTGACTCGCTGCGCTCGGTCGTTCGGCTGCGGCGAGCGGTATCAGCTCACTCA AAGGCGGTAATACGGTTATCCACAGAATCAGGGGATAACGCAGGAAAGAACATGTGAGCAAAAGGCCAGCAAAAGGCCAGGAACC GTAAAAAGGCCGCGTTGCTGGCGTTTTTCCATAGGCTCCGCCCCCCTGACGAGCATCACAAAAATCGACGCTCAAGTCAGAGGTGGC GAAACCCGACAGGACTATAAAGATACCAGGCGTTTCCCCCTGGAAGCTCCCTCGTGCGCTCTCCTGTTCCGACCCTGCCGCTTACCGG ATACCTGTCCGCCTTTCTCCCTTCGGGAAGCGTGGCGCTTTCTCATAGCTCACGCTGTAGGTATCTCAGTTCGGTGTAGGTCGTTCGCT

CCAAGCTGGGCTGTGTGCACGAACCCCCCGTTCAGCCCGACCGCTGCGCCTTATCCGGTAACTATCGTCTTGAGTCCAACCCGGTAAG ACACGACTTATCGCCACTGGCAGCAGCCACTGGTAACAGGATTAGCAGAGCGAGGTATGTAGGCGGTGCTACAGAGTTCTTGAAGT GGTGGCCTAACTACGGCTACACTAGAAGAACAGTATTTGGTATCTGCGCTCTGCTGAAGCCAGTTACCTTCGGAAAAAGAGTTGGTA GCTCTTGATCCGGCAAACAAACCACCGCTGGTAGCGGTGGTTTTTTTGTTTGCAAGCAGCAGATTACGCGCAGAAAAAAAGGATCTC AAGAAGATCCTTTGATCTTTTCTACGGGGTCTGACGCTCAGTGGAACGAAAACTCACGTTAAGGGATTTTGGTCATGAGATTATCAAA AAGGATCTTCACCTAGATCCTTTTAAATTAAAAATGAAGTTTTAAATCAATCTAAAGTATATATGAGTAAACTTGGTCTGACAGTTACC AATGCTTAATCAGTGAGGCACCTATCTCAGCGATCTGTCTATTTCGTTCATCCATAGTTGCCTGACTCCCCGTCGTGTAGATAACTACG ATACGGGAGGGCTTACCATCTGGCCCCAGTGCTGCAATGATACCGCGAGACCCACGCTCACCGGCTCCAGATTTATCAGCAATAAAC CAGCCAGCCGGAAGGGCCGAGCGCAGAAGTGGTCCTGCAACTTTATCCGCCTCCATCCAGTCTATTAATTGTTGCCGGGAAGCTAGA GTAAGTAGTTCGCCAGTTAATAGTTTGCGCAACGTTGTTGCCATTGCTACAGGCATCGTGGTGTCACGCTCGTCGTTTGGTATGGCTT CATTCAGCTCCGGTTCCCAACGATCAAGGCGAGTTACATGATCCCCCATGTTGTGCAAAAAAGCGGTTAGCTCCTTCGGTCCTCCGAT CGTTGTCAGAAGTAAGTTGGCCGCAGTGTTATCACTCATGGTTATGGCAGCACTGCATAATTCTCTTACTGTCATGCCATCCGTAAGAT GCTTTTCTGTGACTGGTGAGTACTCAACCAAGTCATTCTGAGAATAGTGTATGCGGCGACCGAGTTGCTCTTGCCCGGCGTCAATACG GGATAATACCGCGCCACATAGCAGAACTTTAAAAGTGCTCATCATTGGAAAACGTTCTTCGGGGCGAAAACTCTCAAGGATCTTACC GCTGTTGAGATCCAGTTCGATGTAACCCACTCGTGCACCCAACTGATCTTCAGCATCTTTTACTTTCACCAGCGTTTCTGGGTGAGCAA AAACAGGAAGGCAAAATGCCGCAAAAAAGGGAATAAGGGCGACACGGAAATGTTGAATACTCATACTCTTCCTTTTTCAATATTATT GAAGCATTTATCAGGGTTATTGTCTCATGAGCGGATACATATTTGAATGTATTTAGAAAAATAAACAAATAGGGGTTCCGCGCACATT TCCCCGAAAAGTGCCACCTGACGTC

Humanized E protein expression plasmid sequence:

GACGGATCGGGAGATCTCCCGATCCCCTATGGTGCACTCTCAGTACAATCTGCTCTGATGCCGCATAGTTAAGCCAGTATCTGCTCCC TGCTTGTGTGTTGGAGGTCGCTGAGTAGTGCGCGAGCAAAATTTAAGCTACAACAAGGCAAGGCTTGACCGACAATTGCATGAAGA ATCTGCTTAGGGTTAGGCGTTTTGCGCTGCTTCGCGATGTACGGGCCAGATATACGCGTTGACATTGATTATTGACTAGTTATTAATA GTAATCAATTACGGGGTCATTAGTTCATAGCCCATATATGGAGTTCCGCGTTACATAACTTACGGTAAATGGCCCGCCTGGCTGACCG CCCAACGACCCCCGCCCATTGACGTCAATAATGACGTATGTTCCCATAGTAACGCCAATAGGGACTTTCCATTGACGTCAATGGGTGG AGTATTTACGGTAAACTGCCCACTTGGCAGTACATCAAGTGTATCATATGCCAAGTACGCCCCCTATTGACGTCAATGACGGTAAATG GCCCGCCTGGCATTATGCCCAGTACATGACCTTATGGGACTTTCCTACTTGGCAGTACATCTACGTATTAGTCATCGCTATTACCATGG TGATGCGGTTTTGGCAGTACATCAATGGGCGTGGATAGCGGTTTGACTCACGGGGATTTCCAAGTCTCCACCCCATTGACGTCAATG GGAGTTTGTTTTGGCACCAAAATCAACGGGACTTTCCAAAATGTCGTAACAACTCCGCCCCATTGACGCAAATGGGCGGTAGGCGTG TACGGTGGGAGGTCTATATAAGCAGAGCTCTCTGGCTAACTAGAGAACCCACTGCTTACTGGCTTATCGAAATTAATACGACTCACTA TAGGGAGACCCAAGCTGGCTAGCGCAGCATCTTCAGCTCTTGGGTTTAGCAAAAGTTCATCTCCTTCTGCATCCTTAACTGAGAATGA GCTTTTGTGGGAGCCCACACCAGTCAAGTTGGATTTGAACCCAGCTGCTCTGTACAAGCTTGGTACCGCCACCATGTACAGCTTCGTG AGCGAGGAGACCGGCACCCTGATCGTGAACAGCGTGCTGCTGTTCCTGGCCTTCGTGGTGTTCCTGCTGGTGACCCTGGCCATCCTG

ACCGCCCTGAGGCTGTGCGCCTACTGCTGCAACATCGTGAACGTGAGCCTGGTGAAGCCCAGCTTCTACGTGTACAGCAGGGTGAAG AACCTGAACAGCAGCAGGGTGCCCGACCTGCTGGTGTAATCTAGAGGGCCCGTTTAAACCCGCTGATCAGCCTCGACTGTGCCTTCT AGTTGCCAGCCATCTGTTGTTTGCCCCTCCCCCGTGCCTTCCTTGACCCTGGAAGGTGCCACTCCCACTGTCCTTTCCTAATAAAATGA GGAAATTGCATCGCATTGTCTGAGTAGGTGTCATTCTATTCTGGGGGGTGGGGTGGGGCAGGACAGCAAGGGGGAGGATTGGGAA GACAATAGCAGGCATGCTGGGGATGCGGTGGGCTCTATGGCTTCTGAGGCGGAAAGAACCAGCTGGGGCTCTAGGGGGTATCCCC ACGCGCCCTGTAGCGGCGCATTAAGCGCGGCGGGTGTGGTGGTTACGCGCAGCGTGACCGCTACACTTGCCAGCGCCCTAGCGCCC GCTCCTTTCGCTTTCTTCCCTTCCTTTCTCGCCACGTTCGCCGGCTTTCCCCGTCAAGCTCTAAATCGGGGGCTCCCTTTAGGGTTCCGA TTTAGTGCTTTACGGCACCTCGACCCCAAAAAACTTGATTAGGGTGATGGTTCACGTAGTGGGCCATCGCCCTGATAGACGGTTTTTC GCCCTTTGACGTTGGAGTCCACGTTCTTTAATAGTGGACTCTTGTTCCAAACTGGAACAACACTCAACCCTATCTCGGTCTATTCTTTTG ATTTATAAGGGATTTTGCCGATTTCGGCCTATTGGTTAAAAAATGAGCTGATTTAACAAAAATTTAACGCGAATTAATTCTGTGGAAT GTGTGTCAGTTAGGGTGTGGAAAGTCCCCAGGCTCCCCAGCAGGCAGAAGTATGCAAAGCATGCATCTCAATTAGTCAGCAACCAG GTGTGGAAAGTCCCCAGGCTCCCCAGCAGGCAGAAGTATGCAAAGCATGCATCTCAATTAGTCAGCAACCATAGTCCCGCCCCTAAC TCCGCCCATCCCGCCCCTAACTCCGCCCAGTTCCGCCCATTCTCCGCCCCATGGCTGACTAATTTTTTTTATTTATGCAGAGGCCGAGGC CGCCTCTGCCTCTGAGCTATTCCAGAAGTAGTGAGGAGGCTTTTTTGGAGGCCTAGGCTTTTGCAAAAAGCTCCCGGGAGCTTGTATA TCCATTTTCGGATCTGATCAAGAGACAGGATGAGGATCGTTTCGCATGATTGAACAAGATGGATTGCACGCAGGTTCTCCGGCCGCT TGGGTGGAGAGGCTATTCGGCTATGACTGGGCACAACAGACAATCGGCTGCTCTGATGCCGCCGTGTTCCGGCTGTCAGCGCAGGG GCGCCCGGTTCTTTTTGTCAAGACCGACCTGTCCGGTGCCCTGAATGAACTGCAGGACGAGGCAGCGCGGCTATCGTGGCTGGCCAC GACGGGCGTTCCTTGCGCAGCTGTGCTCGACGTTGTCACTGAAGCGGGAAGGGACTGGCTGCTATTGGGCGAAGTGCCGGGGCAG GATCTCCTGTCATCTCACCTTGCTCCTGCCGAGAAAGTATCCATCATGGCTGATGCAATGCGGCGGCTGCATACGCTTGATCCGGCTA CCTGCCCATTCGACCACCAAGCGAAACATCGCATCGAGCGAGCACGTACTCGGATGGAAGCCGGTCTTGTCGATCAGGATGATCTGG ACGAAGAGCATCAGGGGCTCGCGCCAGCCGAACTGTTCGCCAGGCTCAAGGCGCGCATGCCCGACGGCGAGGATCTCGTCGTGACC CATGGCGATGCCTGCTTGCCGAATATCATGGTGGAAAATGGCCGCTTTTCTGGATTCATCGACTGTGGCCGGCTGGGTGTGGCGGAC CGCTATCAGGACATAGCGTTGGCTACCCGTGATATTGCTGAAGAGCTTGGCGGCGAATGGGCTGACCGCTTCCTCGTGCTTTACGGT ATCGCCGCTCCCGATTCGCAGCGCATCGCCTTCTATCGCCTTCTTGACGAGTTCTTCTGAGCGGGACTCTGGGGTTCGAAATGACCGA CCAAGCGACGCCCAACCTGCCATCACGAGATTTCGATTCCACCGCCGCCTTCTATGAAAGGTTGGGCTTCGGAATCGTTTTCCGGGAC GCCGGCTGGATGATCCTCCAGCGCGGGGATCTCATGCTGGAGTTCTTCGCCCACCCCAACTTGTTTATTGCAGCTTATAATGGTTACA AATAAAGCAATAGCATCACAAATTTCACAAATAAAGCATTTTTTTCACTGCATTCTAGTTGTGGTTTGTCCAAACTCATCAATGTATCTT ATCATGTCTGTATACCGTCGACCTCTAGCTAGAGCTTGGCGTAATCATGGTCATAGCTGTTTCCTGTGTGAAATTGTTATCCGCTCACA ATTCCACACAACATACGAGCCGGAAGCATAAAGTGTAAAGCCTGGGGTGCCTAATGAGTGAGCTAACTCACATTAATTGCGTTGCGC TCACTGCCCGCTTTCCAGTCGGGAAACCTGTCGTGCCAGCTGCATTAATGAATCGGCCAACGCGCGGGGAGAGGCGGTTTGCGTATT GGGCGCTCTTCCGCTTCCTCGCTCACTGACTCGCTGCGCTCGGTCGTTCGGCTGCGGCGAGCGGTATCAGCTCACTCAAAGGCGGTA ATACGGTTATCCACAGAATCAGGGGATAACGCAGGAAAGAACATGTGAGCAAAAGGCCAGCAAAAGGCCAGGAACCGTAAAAAGG CCGCGTTGCTGGCGTTTTTCCATAGGCTCCGCCCCCCTGACGAGCATCACAAAAATCGACGCTCAAGTCAGAGGTGGCGAAACCCGA CAGGACTATAAAGATACCAGGCGTTTCCCCCTGGAAGCTCCCTCGTGCGCTCTCCTGTTCCGACCCTGCCGCTTACCGGATACCTGTCC

GCCTTTCTCCCTTCGGGAAGCGTGGCGCTTTCTCATAGCTCACGCTGTAGGTATCTCAGTTCGGTGTAGGTCGTTCGCTCCAAGCTGG GCTGTGTGCACGAACCCCCCGTTCAGCCCGACCGCTGCGCCTTATCCGGTAACTATCGTCTTGAGTCCAACCCGGTAAGACACGACTT ATCGCCACTGGCAGCAGCCACTGGTAACAGGATTAGCAGAGCGAGGTATGTAGGCGGTGCTACAGAGTTCTTGAAGTGGTGGCCTA ACTACGGCTACACTAGAAGAACAGTATTTGGTATCTGCGCTCTGCTGAAGCCAGTTACCTTCGGAAAAAGAGTTGGTAGCTCTTGATC CGGCAAACAAACCACCGCTGGTAGCGGTGGTTTTTTTGTTTGCAAGCAGCAGATTACGCGCAGAAAAAAAGGATCTCAAGAAGATCC TTTGATCTTTTCTACGGGGTCTGACGCTCAGTGGAACGAAAACTCACGTTAAGGGATTTTGGTCATGAGATTATCAAAAAGGATCTTC ACCTAGATCCTTTTAAATTAAAAATGAAGTTTTAAATCAATCTAAAGTATATATGAGTAAACTTGGTCTGACAGTTACCAATGCTTAAT CAGTGAGGCACCTATCTCAGCGATCTGTCTATTTCGTTCATCCATAGTTGCCTGACTCCCCGTCGTGTAGATAACTACGATACGGGAG GGCTTACCATCTGGCCCCAGTGCTGCAATGATACCGCGAGACCCACGCTCACCGGCTCCAGATTTATCAGCAATAAACCAGCCAGCCG GAAGGGCCGAGCGCAGAAGTGGTCCTGCAACTTTATCCGCCTCCATCCAGTCTATTAATTGTTGCCGGGAAGCTAGAGTAAGTAGTT CGCCAGTTAATAGTTTGCGCAACGTTGTTGCCATTGCTACAGGCATCGTGGTGTCACGCTCGTCGTTTGGTATGGCTTCATTCAGCTCC GGTTCCCAACGATCAAGGCGAGTTACATGATCCCCCATGTTGTGCAAAAAAGCGGTTAGCTCCTTCGGTCCTCCGATCGTTGTCAGAA GTAAGTTGGCCGCAGTGTTATCACTCATGGTTATGGCAGCACTGCATAATTCTCTTACTGTCATGCCATCCGTAAGATGCTTTTCTGTG ACTGGTGAGTACTCAACCAAGTCATTCTGAGAATAGTGTATGCGGCGACCGAGTTGCTCTTGCCCGGCGTCAATACGGGATAATACC GCGCCACATAGCAGAACTTTAAAAGTGCTCATCATTGGAAAACGTTCTTCGGGGCGAAAACTCTCAAGGATCTTACCGCTGTTGAGAT CCAGTTCGATGTAACCCACTCGTGCACCCAACTGATCTTCAGCATCTTTTACTTTCACCAGCGTTTCTGGGTGAGCAAAAACAGGAAG GCAAAATGCCGCAAAAAAGGGAATAAGGGCGACACGGAAATGTTGAATACTCATACTCTTCCTTTTTCAATATTATTGAAGCATTTAT CAGGGTTATTGTCTCATGAGCGGATACATATTTGAATGTATTTAGAAAAATAAACAAATAGGGGTTCCGCGCACATTTCCCCGAAAAG TGCCACCTGACGTC
